# Infrared Thermography in the Study of Animals’ Emotional Responses: A Critical Review

**DOI:** 10.3390/ani11092510

**Published:** 2021-08-26

**Authors:** Tiziano Travain, Paola Valsecchi

**Affiliations:** Dipartimento di Scienze Chimiche, della Vita, e della Sostenibilità Ambientale, Università degli Studi di Parma, Parco Area delle Scienze 17/A, 43124 Parma, Italy; paolamaria.valsecchi@unipr.it

**Keywords:** infrared thermography, animal emotions, stress, pets, husbandry system, laboratory animals

## Abstract

**Simple Summary:**

Assessing animal welfare has proven to be a challenging task with important consequences for their management. In the last few years, infrared thermography has gained increasing scientific consensus as a method to analyze emotional reactions to different stimuli in different taxa. This review aims to explore particularly the use of infrared thermography in the assessment of animals’ emotions, mainly focusing on pets, laboratory, and husbandry animals. If properly used, this technique has proven to be a noninvasive, reliable method to identify emotional activations.

**Abstract:**

Whether animals have emotions was historically a long-lasting question but, today, nobody disputes that they do. However, how to assess them and how to guarantee animals their welfare have become important research topics in the last 20 years. Infrared thermography (IRT) is a method to record the electromagnetic radiation emitted by bodies. It can indirectly assess sympathetic and parasympathetic activity via the modification of temperature of different body areas, caused by different phenomena such as stress-induced hyperthermia or variation in blood flow. Compared to other emotional activation assessment methods, IRT has the advantage of being noninvasive, allowing use without the risk of influencing animals’ behavior or physiological responses. This review describes general principles of IRT functioning, as well as its applications in studies regarding emotional reactions of domestic animals, with a brief section dedicated to the experiments on wildlife; it analyzes potentialities and possible flaws, confronting the results obtained in different taxa, and discusses further opportunities for IRT in studies about animal emotions.

## 1. Introduction

### 1.1. Basic Notions of Infrared Thermography

Infrared thermography (IRT) is a remote and noninvasive method to assess the surface temperature of a body through the electromagnetic radiation emitted by any object with a temperature above absolute zero (i.e., 0 K, −273.15 °C). Both the total intensity of radiation and the intensity at any particular wavelength emitted by objects are dependent on their surface and, at high temperatures, correspond to short wavelengths [1]. Inasmuch as the sun’s surface temperature is about 5800 K and its wavelength peak is about 500 nm, it is clear why the visual spectrum of many animals (humans included) is between 390 and 700 nm [2]. Furthermore, items on the earth’s surface emit radiation with a wavelength peak in the region of 10 μm, invisible to human eyes, although it can be experienced as heat (e.g., 300 K, circa 27 °C, means a 9.5 μm wavelength peak). This means that we can see objects only because they reflect sunlight, while their own emitted radiation wavelength peak falls into the infrared radiation spectrum (700 nm–1 mm), which is why we refer to detecting it as infrared thermography [1].

Using IRT to measure surface temperatures of objects requires appropriate tools, usually referred to as infrared thermal (video) cameras, and the consideration of several technical factors: emissivity, observation angle, and distance. 

**Emissivity:** A black body is an ideal item that absorbs all the incident electromagnetic radiation without reflecting it, and it re-emits all received energy regardless of the wavelength because of the law of conservation of energy. Artificial or natural black bodies do not exist. The surface of every real body limits the intensity of the radiation and emits a fraction of the possible radiation at a given wavelength, and this value is called emissivity; it ranges between 0 and 1 (theoretical value of a black body). Experimental measurements of biological tissues in the range of 9–11 μm have shown that absorptivity and emissivity are between 0.9 and 0.97 regardless of the observed color of the surface [1,3].

**Observation angle**: Electromagnetic radiation travels in straight lines; therefore, measuring the intensity of thermal radiation using a very acute angle will reduce the received radiation, and the item will appear cooler than it really is. Even if, generally, the effect of a very acute observation angle is negligible when analyzing uneven surfaces, up to an angle of 10°, it is important to note that, from 30° to 0°, there is an important decrement in received radiation, and eventually none is received at 0° [1]. Consequently, the best position from which to measure electromagnetic radiation is perpendicular to the emitting source (e.g., [4]). For clarity about perpendicularity, Ijichi and colleagues noted that, when IRT images were taken from 90° to the eye, nasal plane, and sagittal plane on horses’ faces, the recorded values significantly varied across the three points of measurement. To identify which point should be considered optimal for the study of arousal, stress, and pain, they exposed horses to a novel object, as a stressful event, and they found a significant positive correlation between eye temperature taken from 90° to the sagittal plane and heart rate variability. Therefore, they suggest that this is the optimal position [5].

**Distance:** The intensity of radiation follows the inverse-square law (the intensity is inversely proportional to the square of the distance from the source), and the material through which the radiation passes may affect transmission. For example, gases absorb certain wavelengths and they may scatter radiation, but disturbances from atmospheric gases are negligible under 10 m [1]. 

Today, IRT has many different applications both in the field of applied sciences (e.g., engineering, material science, optics, or physics) and in the field of biological, medical, and veterinary sciences [6]. For example, it can be used to detect inflammatory conditions, to assess how musculoskeletal systems cope with training in horses, to facilitate diagnosis of some cattle diseases such as bovine viral diarrhea or hoof-and-mouth disease, and to identify rabies-infected wild raccoons [7,8,9].

### 1.2. What Can Infrared Thermography Detect about Emotions?

Body temperature depends on numerous factors; food deprivation will decrease metabolic heat production to preserve energy [10], while bacteria invading the organism can act as exogenous pyrogens through the activation of the immune system (so-called ‘fever’) [11]. Emotions can also alter body temperature since they are responses to external or internal events of a particular significance for the organism (i.e., emotional fever or stress-induced hyperthermia). Emotions are defined as events of short duration characterized by universal facial expressions, phylogenetic continuity of expressed behaviors, physiological activation patterns, and unconscious stimuli evaluation mechanisms [12,13]. At least the basic emotions are widespread across all mammals and birds [14,15] and are divided according to their valence in negative and positive emotions. Negative emotions such as fear and anger play a key role in survival, inducing specific actions to avoid dangerous situations or to cope with stressors through sympathetic nervous system activation [16,17,18], while positive emotions such as joy stimulate curiosity and facilitate social bonding, through the activation of the parasympathetic nervous system [19,20].

On one hand, stressors cause sympathetic nervous system activation and, therefore, alteration in hypothalamic–pituitary–adrenal (HPA) axis activity and, consequently, in glucocorticoid secretion by the adrenal cortex [21]. This could lead to an activation of the sympathetic–adrenal–medullary (SAM) system, which is responsible for ‘fight or flight’ responses, thanks to the production of catecholamines from the adrenal medulla [22]. The whole process causes an increment in internal body temperature called ‘stress-induced hyperthermia’ (SIH), and it is a common response to acute stress or to repeated stressing episodes [23]. Interestingly, even if stress-induced hyperthermia is not a form of fever, SIH and fever have overlapping activation pathways that likely include neurons in the dorsomedial hypothalamus [24,25]. On the other hand, the parasympathetic nervous system controls ‘rest and digest’ functions, which cause, among others, heart rate reduction, bronchoconstriction, vasodilation, and miosis [26].

Internal temperature changes, ‘rest and digest’, and ‘fight or flight’, reactions cause consequent changes in external temperature. For example, during a ‘fight or flight’ response, peripheral areas have reduced blood flow in order to avoid severe blood loss in case of injury and to reallocate blood where it is most needed, resulting in a reduced external temperature in these areas [27]. Therefore, detecting these changes means detecting autonomic nervous system activation. Infrared thermography is particularly suitable for this task, especially when targeting body areas mainly used for thermal regulation, without a massive coverage of hair or plumage, which are not frequently wet (e.g., dogs’ noses). Additionally, eyes are a particularly appropriate zone because of the rich capillary beds that rapidly respond to changes in blood flow present on the small areas around the posterior border of the eyelid and on the lacrimal caruncle [28,29].

### 1.3. Literature Review

Although reviews about the use of infrared thermography in animals already exist (e.g., [1,30,31,32]), this is the first one concerning specifically the study of animals’ emotions with the aid of IRT.

**Figure 1:**
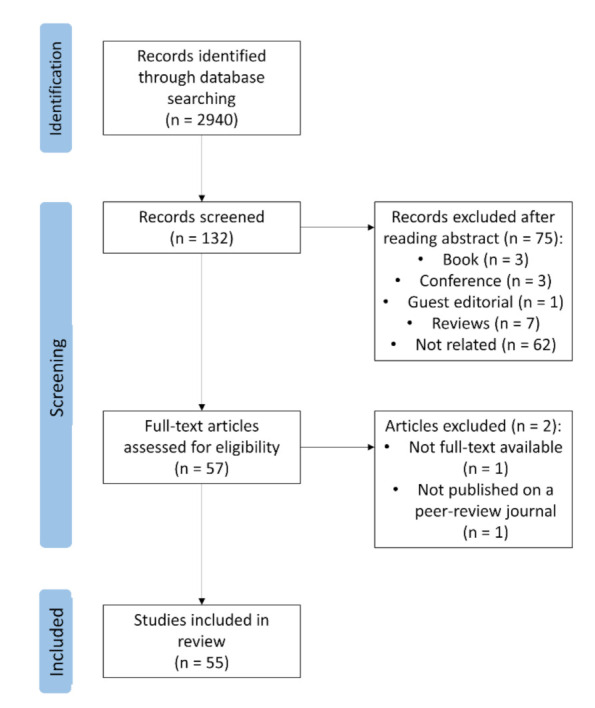
Flow chart for selection of studies.

The literature searches via Google Scholar, Web of Sciences, and Scopus included only studies published in peer-reviewed journals. The process started by cataloging all articles containing ‘infrared thermography’, ‘emotion’, and ‘animals’ or at least one animal species in the keywords and/or in the title. The first screening, by the title, resulted in a list of 132 articles. Subsequently, given the aim of this review, irrelevant articles were removed by examining the abstracts. For example, various veterinary science articles about clinical applications of IRT that marginally mentioned animal welfare were included in this preliminary database. Figure 1 shows the article selection process followed in this review.

The drafting of this review followed a taxonomic criterion, with the analyzed studies divided according to the order to which the studied species belonged. In Appendix A, Table A1 displays the species and the stimuli for the selected articles. According to the results, 52 out of 55 papers reported a temperature change in response to the given stimulus. In 19 out of 52 studies (36.54%), the authors’ hypothesis was, at least partially, confirmed; in two out of 52 (3.85%), the authors’ hypothesis was rejected; in four out of 52 (7.69%), the hypothesis was not related to temperature changes or IRT was used as a tool to verify different hypothesis; in 27 out of 52 (51.92%), no clearly hypothesis was stated, but the authors rather referred to the existing literature or supposed a change in temperature without giving predictions about the direction of this change.

## 2. Applications

### 2.1. Nonhuman Primates

Seven papers aimed at assessing the utility of IRT to study emotions in nonhuman primates have been published since 2005: three on rhesus macaques (*Macaca mulatta*), two on chimpanzees (*Pan troglodytes*), one on common marmosets (*Callithrix jacchus*)*,* and one involving both monkey and ape taxa.

With the exception of the work by Ioannou and coworkers [33], all studies carried out to investigate rhesus macaques’ emotional reactions featured animals restrained on a dedicated primate chair and exposed to different stimuli. The first study was published in 2005 [34], and it had four female monkeys presented with a negative stimulus, with a person standing 1 m from them for 3 min, while nose temperature was assessed. Compared to a previous control phase, the expected temperature decrease happened within 10–30 s and continued while monkeys expressed faces with silent bared teeth, staring open mouth, and lip smacking, behaviors related to negative emotions, for 3–4 min. Although being restrained to the chair is a stressful event per se, it is unlikely that it caused the temperature changes because the monkeys were already habituated to the use of the device and had been familiar with it for more than 2 years. Nevertheless, the authors admitted that restraining the monkeys could have been a limitation. In another study, three rhesus macaques were exposed to trials of negative social stimuli (a clip of a raging, unfamiliar individual, two shorter clips of an aggressive threat, and a close-up shot of a scream by unfamiliar individuals) and a neutral stimulus (a short clip of a so called ‘coo’, a typical social macaque call). The aim was to confirm the observations from the previous study with bidimensional stimuli, commonly used in studies on animal emotions, and to assess temperature changes in response to emotion other than just threats [35]. Macaques’ nose temperature decreased upon receiving negative stimuli, while no changes were detected during the neutral one. The authors argued that these outcomes supported the use of IRT as a tool for detecting emotional reactions in rhesus monkeys and suggested that, even through a digital domain, both facial expression and vocalization can convey the same emotional meaning as a live presenter, despite the use of vocal signals during social interactions being relatively uncommon in rhesus macaques. Ioannou and coworkers [33] conducted an experiment with a toy to play with, food teasing (presenting and moving one mealworm or a cricket in front of the animal, without giving access to it), and food (one mealworm or a cricket to eat) as stimuli. Additionally, monkeys were not restrained in a chair, but they were free to move in an outdoor enclosure. In addition to the nose, the authors scanned other facial areas: nose tip, maxillary area, and periorbital region. During feeding, the temperature in all target areas decreased, whereas, during teasing, it increased only in the periorbital region, and no changes occurred while playing with the toy. Increments during food teasing could have been caused by increased blood perfusion during anger or by prolonged activity of muscles around the eyes, because subjects were constantly following the treat.

Kano and coworkers performed two experiments on chimpanzees, exposing the animals to audio or visual stimuli while assessing nose temperature [36]. In the audio experiment, stimuli were fighting vocalizations emitted by group mate individuals or display calls emitted by an allospecific individual (orangutan, *Pongo* sp.). The video experiment featured conspecifics fighting (with screaming and barking vocalizations) or conspecifics resting (with natural background noises). In both experiments, no sound in the testing room or a blank screen (with no sound) served as a control situation. Nose temperature dropped more during the fighting conditions than in the other two situations, both in the video and in the audio experiments. Interestingly, the temperature drop occurred despite the fact that the chimpanzees were visibly excited (e.g., hitting the monitor or swaying the body, but no locomotor activity) during the fighting condition, supporting the hypothesis that activation of the sympathetic neural system caused both behavioral and physiological changes. The second study on chimpanzees used IRT for the first time on a wild population of 14 eastern chimpanzees (*Pan troglodytes schweinfurthii*) habituated to the presence of human observers [37]. Experimenters scanned nose and ear areas of chimpanzees during episodes of spontaneous vocalization emission (screams/whimpers, pant hoots, aggressive barks, travel and resting hoos, grunts, and pant-grunts). Results showed that aversive calls caused a larger decrease in nose temperature, while neutral calls did not; on the contrary, neutral calls caused an increment in ear temperature with no significant change caused by aversive calls. The authors concluded that changes in nasal temperature might be a byproduct of either increased breathing in a stressful situation or subcutaneous vascular constriction, while the increment in ear temperature might happen because of an increased blood flow in the area to increase auditory efficiency. This experiment had two potential pitfalls: the lack of relevant social interactions (e.g., physical aggression or a mother running to protect her offspring), because these have concurrent physical activity, which makes the use of IRT difficult, and the difficulty to assign every change to a precise event, which is a particularly important task to assess the key stimuli triggering temperature changes. Nevertheless, this experiment was important because it showed that IRT is viable even with primates in a wild context with limited time to gather data of target areas.

The experiment of Chotard and colleagues [38] involved three different monkey taxa (common marmoset, *Callithrix jacchus*; Colombian white-faced capuchin, *Cebus capucinus*; rhesus macaque) and two ape taxa (Müller’s gibbon, *Hylobates muelleri*; western lowland gorilla, *Gorilla gorilla gorilla*). Eight animals were stimulated with toys and teasing as positive and negative stimuli, respectively; the Müller’s gibbon received only the toy interaction, and a gorilla received tickling and food delay (with a second one experiencing only food delay). The periorbital area, nose bridge, nose tip, and upper lip were the measured areas. Positive stimuli caused a temperature decrease in the nose tip and an increase in the periorbital area, but no change for the nasal bridge and upper lip. However, individuals of all species did not uniformly show these changes; in fact, removing data from gorillas, no differences emerged among the monkey species. Negative stimuli caused an increase in upper lip, confirmed even when removing the gorilla data from the sample. Lastly, when common marmosets were exposed to different positive (preferred food and playback of food calls) and negative (playback of aggressive vocalizations and food teasing) stimuli, nasal temperature showed a different pattern of changes. As the authors supposed, in the negative conditions, there was a decrease in nasal temperature, with females having a more pronounced change than males, and, in the positive conditions, males’ temperature increased while females had a decrease after the playback of food calls. These reactions and sex differences were consistent with naturalistic observations, with higher food motivation and competition among females and stronger reactions to separation from group members in males; therefore, the results supported a negative correlation between arousal and nasal temperature [39].

### 2.2. Even-Toed Ungulates

Since even-toed ungulates are massively farmed, studies on cattle, pigs, sheep, and goats focused almost entirely on stress, fear, and negative situations with the aim of improving general animal conditions and welfare; 11 papers aimed to study emotional reactions with IRT in even-toed ungulates: five on cattle (*Bos taurus*), two on Caprinae, and four on pigs (*Sus scrofa domesticus*).

In 2006, two studies by Warriss and colleagues explored the thermal reaction of pigs when transported to the slaughterhouse. In a sample of 384 pigs, the authors measured the postmortem temperature of the inner surface of the ear and of the blood lost at exsanguination, and they found a positive correlation with the level of creatine kinase in the blood. According to the authors’ interpretation, the significant correlation found suggested that hotter pigs might have been suffering more stress during transportation and slaughter than others [40]. A second study was carried out with 417 pigs transported to the slaughterhouse in pens with fan-assisted or natural ventilation. The authors collected the temperature of blood lost at exsanguination and of the inner surface of the ear, the blood composition, the muscle glycogen concentration, and the position on the truck. Ear temperature increased from the back to front of the truck, which may be explained by the direction of air flow. However, the differences in ear and blood temperature in pigs with or without fan-assisted ventilation led the authors to conclude that fans were not sufficient to remove body-generated heat and guarantee increased welfare during transport. Lastly, pigs carried with fans had no changes in their blood composition or muscle glycogen levels compared to those carried without fans; therefore, they experienced the same levels of stress [41]. More recently, a study examined the effects of different environmental enrichments for 64 weaned piglets divided into two different groups: the first formed of littermates and the other with piglets from different litters [42]. Experimenters further divided the sample into four subgroups subjected to different stimuli: suspended ropes, bottles aromatized with strawberry essence, pet toys and balls, and a control situation with no enrichments. Physical activity and temperatures of the lacrimal caruncle, auricular pavilion, and nose area were assessed 30 and 90 min after the exposure, and then every 24 h (last collection at 120 h). The temperature changes among the different groups were not sufficiently clear to identify the effects of the different stimuli 24 and 48 h after the beginning, and the authors concluded that IRT might not be sensitive enough. However, the auricular pavilion and nose area temperature of the pigs in the group of unfamiliar individuals was higher than the corresponding temperatures of pigs in the littermate group. This likely happened because pigs in the unfamiliar group needed to establish a new social hierarchy, and this determined an increased physical activity and agonistic interactions. 

Because, in intensive farming systems, pigs live in small areas, in close contact with each other, the study by Boileau and colleagues [43], aimed at testing if skin temperature correlates with variations in behavioral and physiological responses during agonistic encounters, was particularly interesting. The authors measured back skin temperature while pigs were engaged in agonistic dyadic encounters in a 2.9 × 3.8 m arena. Minimum, maximum, and average temperature was assessed for five different events: start of the contest, the agonistic phase, the retreat of the loser, the post-retreat phase, and the end of the contest (i.e., 1 min after retreat). There were no temperature differences in winners or losers during the different phases, with both animals experiencing a strong decline during the retreat of the loser; if pigs engaged in a fight, this further lowered both animals’ temperature. The number of skin injuries was significantly correlated with body temperature; animals with many skin lesions had larger changes in temperature. The similar temperature patterns in winners and losers, featuring a strong temperature decrease at the contest resolution, occurred even in the contests without fights and, therefore, without skin lesions, confirming that social conflict is a powerful stressor and suggesting that the physical activity was not the primary cause of peripheral temperature changes.

In cattle, the first study investigated whether IRT could detect stress responses [21]. In two different weeks, six cows were subjected to five exogenous treatments via jugular catheter (saline solution; adrenocorticotropic hormone, ACTH; bovine corticotrophin-releasing hormone, bCRH, 20 μg; bCRH, 40 μg; epinephrine) and a social stimulus (isolation), while eye and core temperature, and plasma concentrations of cortisol, ACTH, and non-esterified fatty acids (NEFAs) were assessed 30 and 60 min after the treatment. ACTH and bCRH caused a stress reaction to evaluate which HPA axis functions were involved, while epinephrine was used to determine if the sympathetic nervous system affected eye temperature as it includes functions of pupil dilatation and blood flow constriction in the skin and gut. Catheterization itself caused a rise in eye temperature during the second week. Eye temperature was unaffected by isolation; bCRH and epinephrine levels were higher 30 and 60 min following control and ACTH. Despite the physiological parameter responses being consistent with the stimuli, with the exception of ACTH, the temperature did not change as expected (i.e., dropping with epinephrine and rising with exogenous stimulation of the HPA axis). The unexpected results raised questions about the experimental setup; although the cows were habituated to both human contact and procedures, they might have perceived handling as stressful, causing eye temperature to increase before treatment. Furthermore, 30 and 60 min could have been a too long a time interval to capture significant eye and core temperature changes. However, another relevant hypothesis is that sympathetic activation itself does not produce a stress response, but when stress or fear is present, sympathetic activation enhances the response. Therefore, the exogenous treatments alone would not have been sufficient without a cognitive awareness of the stressor. However, under natural conditions, a fight-or-flight response would occur over several min; therefore, the authors suggested that infusion may be closer to a realistic endogenous epinephrine release after exposure to a stressor stimulus and, indeed, they tried this second approach. Sixteen calves received a jugular infusion of either epinephrine or physiological saline. While eye temperature did not change with the saline infusion, it decreased with the increase in plasma epinephrine concentration as expected, supporting the hypothesis that eye temperature is mediated by the sympathetic component of the ANS [44]. 

The same research group conducted three other experiments on stress and fear reactions in cattle [29,45,46], using various stressors ranging from a mild (restraint) to an intense (disbudding without anesthetic) stimulus. Eye temperature decreased after exposure to the stressors, with the exception of disbudding with anesthetic and restraint. Interestingly, a more stressful and painful stimulus, such as disbudding without anesthetic, caused both a more intense temperature decrease and a longer recovery time, with increasing eye temperature over recovery. The drop in temperature may have been due to sympathetically mediated vasoconstriction that causes blood to flow from the capillary vessels. Consistently, the use of an anesthetic prior to the disbudding eliminated the pain and the temperature drop, but not the subsequent increase in eye temperature. However, the mechanism underlying the involvement of pain in this response is still unknown. In addition to the above findings, one of these experiments demonstrated that it is possible to use IRT to reliably measure respiratory rate (RR), when cows are restrained in a cattle crush. Despite the fact that the stressor did not cause any variation in the assessed parameters, continuous IRT imaging of airflow had good agreement with different methods of measuring RR, proving IRT’s usefulness for future applications in monitoring cattle welfare under husbandry systems [46].

Cannas and colleagues [47] used the voluntary animal approach (VAA) test to assess if IRT could be used to measure a physiological reaction of stress and fear in sheep (*Ovis aries*) caused by handling. A first VAA test was conducted 1 h before the restraint and a second one the day after; they consisted of an unfamiliar experimenter entering the pen and staying silently crouched for 5 min. For the restraint procedure, the handler initially cupped their hand under the jaw of the sheep, and then they grabbed the bony part of the jaw and kept the sheep’s head up. The handler positioned their left knee just behind the sheep’s left shoulder, while their right leg touched the sheep’s side near its left hip. After being restrained in this position for 5 min, the sheep was released in the pen. During restraint, eye temperature was higher than both VAA tests, and, during the second test, the temperature was higher than the first case. Results suggested that the unfamiliar experimenter was perceived as more threatening after the handling procedure, with the restraint having a negative impact on the human–animal relationship. In 2019, Bartolomé and colleagues [48] validated the use of IRT to analyze stress reactions in goats (*Capra aegagrus hircus*) caused by a person walking around the animals, making noise, and waving their arms to guide them to another pen. Respiratory rate and lacrimal caruncle temperature were higher after the stress test than before, and there was a positive correlation between them.

### 2.3. Odd-Toed Ungulates

There are 11 studies assessing odd-toed ungulates’ emotional reactions with IRT, and they focused exclusively on horses. Two studies carried out during jumping competitions [49,50] measured lacrimal caruncle temperature 3 h before the competition, 5 min after the competition, and 3 h after the competition, as well as their correlations with salivary cortisol [49], heart rate, and competition performance, respectively [50]. In both experiments, eye temperature increased during the competition, dropped to baseline levels 3 h after the competition, and was partially affected by horse age and genetic line. Older horses had lower temperature values, being more habituated and less stressed, and the Anglo-Arab genetic line had higher temperature values when compared with the German, Selle Français, and mixed genetic lines. A correlation between temperature and salivary cortisol was only found 3 h after the competition [49]. Heart rate correlated positively with eye temperature before and immediately after the competition, while eye temperature negatively correlated only with competition results immediately after [50]. The authors stated that the rise in heart rate indicated an activation of the HPA axis and a consequent increment in sympatho-adrenal activity, and they suggested a similar physiological base for the temperature increase, considering that the lacrimal caruncle is very sensitive to both pain and stress [49,50].

Aside from jumping competitions, both trot and endurance races were stressful events for horses. Eye temperatures of trotter horses before and after a race varied and correlated to performance; if the initial temperature was higher than the final one, then horses had a worse performance [51]. This suggested that stress levels before the race influenced competition results, and that horses that perceived the race as stressful had worse results. The authors calculated the relative temperature value when physiological stress turned into distress, lowering the race performance and potentially causing negative effects on welfare that corresponded to the final temperature being 0.97% lower than initial temperature. From that point on, horse performance improved. This was confirmed by previous studies showing a connection between HPA axis activation and the intensity of physical activity in horses; during physical activity, the sympatho-adrenal and the HPA axes are active, responding to an acute stress stimulation [52]. As in studies on jumping competitions, older animals had lower eye temperature and differences between initial and final temperature values [51]. Similarly, Redaelli and colleagues [53] tested eight horses during their training for endurance events, in order to assess IRT as a useful noninvasive tool to use for periodic health assessments during races. During trainings with three different intensities, experimenters collected the temperature of the lacrimal caruncle, front crown, rear crown, front pastern, rear pastern, *gluteus*, and *longissimus dorsi*, as well as HR, blood count, and serum cortisol. As expected, all parameters increased after training, eye temperature correlated positively with HR, and crown temperature correlated positively with cortisol. Only HR and white blood cell concentration increased with the intensity of the exercise. Findings suggested that eye and crown temperature might be suitable to detect both physiological stress and horses that are not fit to compete or to continue the competition.

As a source of stress for horses, bridles have received particular attention; both McGreevy and colleagues [54] and Fenner and colleagues [55] evaluated their negative effect on animals. In the first experiment, horses had to wear a double bridle, with or without a cavesson noseband. Results suggested that wearing double bridles and nosebands caused an increase in eye temperature and a decrease in skin temperature. Therefore, wearing double bridles and tight nosebands caused a physiological stress response and could have compromised vascular perfusion. Consequently, the authors concluded that the use of nosebands that cause any constriction of jaw movement should be reconsidered to improve horses’ welfare [54]. In the second experiment, the conditions were an unfastened noseband (UN), a conventional space of two fingers of space available under the noseband (CAUN), a half-conventional space under the noseband with one finger of space (HCAUN), and no space for fingers under the noseband (NAUN), which was expected to trigger an increment in eye temperature. Eventually, during NAUN, eye temperature and HR increased, while natural oral behavior such as chewing, licking, yawning, and swallowing decreased or was absent during HCAUN and NAUN. However, after the removal of the noseband and double bridle, these behaviors were expressed again. Results suggested the existence of a motivation to perform these behaviors and implied that their inhibition placed horses in a state of deprivation, in addition to causing physiological stress responses [55]. Similarly, hair clipping has been considered a stressful activity in horse management. Adult horses that were either compliant (C) or noncompliant (NC) toward this practice experienced a sham clipping (with running clippers, but without active blades) procedure. Behaviorally, NC horses were less relaxed than C horses, and their heart rate was significantly higher only after 10 min of procedure. All horses had an increase in heart rate and peak in salivary cortisol after the sham clipping. Furthermore, according to the authors’ hypothesis, eye temperature increased in all horses during the procedure and decreased immediately after, showing a positive correlation with salivary cortisol. This seemed to suggest that, even if the behavioral response of C and NC horses was different, all horses found the procedure aversive [56].

Additionally, fear-inducing events, such as an unknown noise, triggered increases in lacrimal caruncle temperature in sport horses. After being exposed to the noise of a dropping bottle, horses that subsequently did not approach it again had a higher variation in eye temperature during a fearful situation [57]. Ijichi, Squibb, and coworkers [58,59] tested horses’ reactions when navigating two distinct obstacles whilst handled by the owner or a stranger, and they monitored completion time, eye temperature, HR, HRV, and proactive and reactive behaviors, namely, horses’ movements to avoid the stressors or freeze responses, emotional blunting, and unresponsiveness. Contrary to the hypothesis, the handler did not influence behavioral or physiological responses, and physiological parameters did not correlate with spent time or with level of proactivity. This indicates that some subjects crossed an object they found aversive, and that proactive horses, which seemed more stressed, actually showed similar reactions to reactive individuals. Results raised doubts about the reliability of the behavioral signals commonly used in the equestrian industry to detect horses’ stress.

Interestingly, a study correlated eye temperature, behavior, performance, genetic line, and age of horses attending dressage competitions with the aim of assessing the usefulness of eye temperature for both competition performance analysis and improvement of the Pura Raza Español (PRE) breeding program. Results showed that there was heritability for both eye temperature and performance traits, particularly when analyzing data from the top performers. These findings could lead to the development of IRT as a potential tool involved in the selection of the best animals for dressage performance, even if this would involve selecting the animals with higher eye temperature values (i.e., with higher physiological stress). Therefore, even if IRT is a potential tool for the improvement of PRE breeding, more evidence on its validity, sensitivity, and specificity is still needed [60].

### 2.4. Rodents and Lagomorphs

Millions of rodents and lagomorphs are used in animal testing every year; in particular, mice are commonly used due to their low maintenance cost, ease of handling, and fast reproduction rate (Willis-Owen and Flint 2006). Therefore, it is important to assess their stress levels and emotional conditions in order to ensure their welfare and the absence of factors that could compromise other studies. Similarly to livestock, all reported experiments focused on emotional reactions to negative stimuli, with seven featuring rodents and two featuring lagomorphs.

Vianna and Carrive [27] conditioned six laboratory rats (*Rattus norvegicus domestica*) to a foot shock chamber, while another six were kept as control and subjected to the same procedure but without the shock. After the conditioning, all of them were again exposed to a foot shock chamber, and their temperature, behavior, heart rate, and arterial pressure were assessed. Rats conditioned to receive the shock expressed intense freezing behavior and a drop in tail and paw temperature, which lasted for the entire duration of the exposure. In the control group, the temperature drop was about half the magnitude and duration. Eye, head, and back temperature increased, with no difference between the groups, but the increase in body temperature was slightly higher and delayed in conditioned rats. Ending of exposure was associated with a gradual decrease in body temperature and an increase in tail temperature. Fear and, to some extent, arousal evoked a strong cutaneous vasoconstriction in the tail and paws. This reduction in blood flow might be preparatory for a possible fight or flight reaction, and it might reduce blood loss in case of injury. After the exposure to the stressor, the tail was the main part of the body used for dissipating accumulated internal heat.

An experiment [61] with 26 male domestic mice (*Mus musculus domesticus*) subjected to both open field (OF) and elevated plus maze (EPM) situations showed that tail and eye temperature changed in both experiences, decreasing in the first while increasing in the latter. OF and EPM are unconditioned fear tests exploiting the animals’ natural aversion for unfamiliar open and elevated spaces that are widely used with laboratory rodents [62]. In OF, the initial standard deviation of temperatures and the tail and eye temperature were significantly and positively correlated with distance covered during the first 5 min of testing. In EPM, initial eye temperature was significantly and positively correlated with the percentage of open arm entries, and the correlation between the temperature at the beginning of tests and behavioral responses suggested the predictive value of initial individual temperature. The hypothesis was that interindividual variability in temperature reflected differences in individual metabolism; therefore, more active individuals in the unfamiliar environment could similarly behave in their familiar environment. Animals that were more anxious had a stronger increase in standard deviation of temperatures because a concomitant increase in maximum and decrease in minimum temperature led to an increase in the variability of the individual body temperature. Therefore, the authors supported the idea that this measure is a well-suited indicator of an animal’s emotional responses. 

Similarly, Duparcq and colleagues [63] tried to test mound-building mice (*Mus spicilegus*) in an OF. They revealed an expected and significant increase in whole-body temperature after handling and transfer into the apparatus, and they found that, shortly after handling, fast exploring mice showed significantly lower tail temperature than slow exploring mice, meaning that there could be a stronger sympathetic reactivity in fast explorers than slow ones. Interestingly, this shows that animals with different behavioral reactions may have associated differences in their physiological stress response such as, for example, differential reactivity of the sympathetic nervous system. In the study by Faraji and Metz [64], mice faced both a multidimensional restraint and a vertical exploration with rearing deprivation to record changes in cutaneous head (including eyes), back, and tail temperature after the stressful events. In the multidimensional restraint, mice were inside a transparent Plexiglas tube that maintained them in a standing position. In the rearing deprivation, mice were inside a white PVC pipe that allowed animals to freely move and turn around but prevented rearing on their hind limbs. Restraint stress increased temperature similarly both in males and in females in all body regions. However, only females responded to rearing deprivation with increased cutaneous temperature in the head and back, as well as a reduced thermal response in the tail, and these are the first findings on mouse sex differences recorded with IRT.

In addition to tests explicitly designed to cause fear and/or stress, laboratory animals experience everyday life stressors such as injections or handling. Gjendal and colleagues explored the impact of these kinds of stressors on mice anxiety [65]. In their experiment, 80 male domestic mice were allocated to a control group (C) or subjected to one of the following stressors: 10 min of anesthesia with isoflurane (ISO), handling by the scruff (SCRUF), or intraperitoneal injection (IP). Then, mice performed the elevated plus maze to assess different levels of anxiety. Eye, body, and tail temperature were collected. ISO led to a decrease in maximum eye temperature, average body temperature, and maximum tail base temperature, and it was the only confirmed hypothesis. Animals returned to baseline values within 10 min SCRUF and IP did not cause any variation in temperature. Despite the temperature differences, subsequent mouse behavior in the EPM showed that all groups had a similar anxiety level. According to authors, SCRUF and IP were too mild stimuli to elicit significant temperature variations, and the lack of differences in the EPM results supported this conclusion. Moreover, because anesthesia is known to lower body temperature, temperature increase following ISO drops cannot be imputed to stress-induced hyperthermia. Concluding, the authors analyzed the best spots for temperature analysis. Eyes are a promising area; however, due to their dimensions, it is difficult to obtain high-quality pictures. On the other hand, the tail presents too much variation compared to the body. Lastly, the body itself is reliable and easily accessible. Therefore, average body temperature is ideal for measuring temperature variations in mice.

Until now, applications of IRT on rabbits have been scant. Six rabbits (*Oryctolagus cuniculus*) were exposed to social stress (being in the same cage with an unfamiliar individual for 30 min), a sudden noise, and tonic immobility (being placed on the back for 3 min), with both ear and eye temperature monitored. Temperature dropped during all stress conditions, with the ear having a more pronounced change. This led the authors to conclude that, unlike other animals, the best site to assess stress reactions with IRT in rabbits is the ear skin, due to vasoconstriction processes. Additionally, stress conditions caused a slight increment in corticosterone levels, suggesting HPA axis activation [66]. Jaén-Téllez [67] and colleagues registered the nose, ear, and eye temperature of fattening rabbits in the period before and after a handler held the rabbits in their arms for 1 min, and it slightly increased in the ear and eyes, showing an acute stress response caused by the release of catecholamines in the adrenergic synapses. In the case of the nose, its humidity, which can vary according to the room temperature, can alter the temperature measurement.

It is worth noting that Schraft and Clark [68] managed to use infrared thermography in a field experiment. They exposed free-ranging Merriam’s kangaroo rats (*Dipodomys merriami*) to a predator, a tethered Mojave rattlesnake (*Crotalus scutulatus*), and measured thermic reactions of different body parts. Supporting the authors’ hypothesis, the temperature of the extremities changed, as eye and snout temperature decreased, and hind leg temperature increased. On the other hand, tail base temperature was significantly predicted by ambient temperature, and this could mean that the tail is correlated to a thermoregulatory mechanism. Eye, snout, and hind leg changes were those expected in a ‘fight or flight’ reaction. These different mechanisms are not mutually exclusive and may even interact, with the redistribution of blood amplifying temperature changes. It was unclear if these thermic changes could help rattlesnakes to locate their prey or if they interfere (e.g., ground squirrels, *Spermophilus beecheyi*, use a thermic signal as an anti-predatory strategy against rattlesnakes [69]). Regarding anti-predation strategies, Lecorps and colleagues [70] exposed mice to TMT (2,5-dihydro-2,4,5-trimethylthiazole), a substance isolated from fox feces. Eye and tail temperature were positively correlated and increased within 2 and 4 min, respectively, and eye temperature decreased to baseline 5 min after exposure. An increase in tail temperature after exposure contrasted with the authors’ hypothesis and physiological changes related to fear responses, suggesting that the different physiological stress parameters involved during a fear response can change independently. This might be because TMT is only an indirect threatening cue, as the predator fecal odor does not mean that the predator itself is nearby.

### 2.5. Carnivores

Dogs and cats are widespread companion animals that have shared a long evolutionary history with humans [71], on whom they are highly dependent for both health and care. Actually, in Western culture, they are considered friends or family members; therefore, their emotional wellbeing is an important and studied topic. Eight studies have been carried out on dogs, with only one on cats.

Veterinary examination is a notoriously stressful event for dogs; during an exam, eye temperature significantly increased, as supposed by authors, while returning to baseline values when finished [4,72]. Moreover, because movement rate had a trend inversely proportional to eye temperature, it was possible to suggest that the increase in eye temperature was due to psychogenic stress and not physical activity [4]. Castration is a standard veterinary procedure that is known to cause post-surgery pain in dogs; analyzing behavioral responses, eye temperature, and dogs’ personality (using the Monash Canine Personality Questionnaire—revised version [73]), it emerged that behavioral responses did not correlate with eye temperature, while ‘extravert’ (i.e., dogs that are typically active, excitable, and restless) personality traits did [74]. The authors suggested that these results could indicate a potential use of personality as a clinical tool for assessing individual differences in response to pain. Because arousal caused an increase in eye temperature [4] and pain caused a decrease [29], more extraverted individuals could have had increased arousal, while introverted individuals experienced a pain-induced temperature decline. Interestingly, extraverted individuals also had greater right eye temperature, suggesting a different blood flow in the two hemispheres [74].

As in horses, racing greyhounds’ stress and arousal can be measured with IRT. Eye temperature was collected 10–15 min before and 15 min after a race. Eye temperature after the race was significantly predicted by ambient temperature, humidity, and time spent by dogs at the race meet, and there was a significantly greater increase in the right eye rather than in the left eye. Additionally, higher temperatures were positively correlated with bad results, young age, and light-colored coat. This might suggest a stronger stress response to racing, but it could also be possible that increased eye temperature indicated a higher core body temperature due to physical activity rather than emotional stimulation. The question remained unsolved because the racing rules prevented authors from collecting post-race images more than 15 min from the conclusion, while the time needed for dogs’ core body temperature to return to baseline was about 30 min. The difference between eyes may suggest that the right eye was more sensitive, but the anticlockwise single direction of the races may also have caused the effect. Regarding the time spent at the race event, racing greyhounds were kenneled 30 min before the first race, and, after the first race, handlers continuously entered the kennels to collect dogs and return dogs that had just raced, exposing kenneled dogs to ongoing disturbance and auditory stimuli from outside. Furthermore, considering that animals cannot know when they will race, an anticipation effect might occur every time they are disturbed by the movements of trainers and dogs and they are not taken to the racing ground [75,76]. A less intense stimulus, the approach of a familiar or unfamiliar person, did not elicit any change in eye temperature in dogs. Either a familiar or an unfamiliar person, with a smile or without any particular facial expression, approached 13 dogs and stopped 70 cm from them. The lack of thermal reactions might have been because of the stimulus itself, but also because of the short duration of the experiment, with the entire approach procedure lasting 5 min [77].

Regarding positive stimulation, palatable treats could be considered a sort of reward and may be able to focus the animal’s attention toward the giver. Consequently, when 19 dogs received palatable food, eye temperature was higher compared to a baseline measurement, and the behavior was consistent with a positive activation. Subsequently, eye temperature returned to baseline values. This was in line with authors’ predictions; however, as shown above, eye temperatures have also been shown to raise when dogs are negatively stimulated. Therefore, although IRT might be a useful tool in assessing arousal emotional states in dogs, it fails to discriminate emotional states, whose interpretation still has to depend on behavioral indices or supplementary physiological parameters [78]. Riemer and coworkers measured ear temperature in dogs during a brief separation from their owner and found that it decreased during separation and increased when a person (either owner or stranger) was present [79]. Although these results indicate that IRT could be promising as a noninvasive remote stress assessment tool, ears were often difficult to use (if not completely inaccessible) because of the different hair and ear structure of many dog breeds.

The only existing study on cats investigated the association between eye temperature and personality [80]. Cats were hosted in cat rehoming centers, and their eye temperature was measured when they were quiet in their pen, straight after the personality profiling (assessed with feline temperament profile, FTP [81,82]) and 1 h after the FTP. In line with the authors’ prediction, temperature was negatively correlated with FTP scores (predicting cats with more stress responses) and was higher in older cats and in those singly housed. The authors suggested that personality might be a predictor for stress sensitivity in cats and that these findings may improve adoption success rate, because more knowledge about the personality and stress sensitivity of cats by the owner could reduce avoidance and aggressive behaviors.

### 2.6. Gallinaceous and Passerine

As previously stated, livestock welfare and management are topics that have seen growing interest in the public opinion, because of a more developed sensibility for animal conditions under husbandry systems. This public concern has led to various studies about common stressful situations occurring during chickens’ lifetimes and reactions to different environmental conditions. At present, there have been seven studies regarding gallinaceous and one about passerine.

In an interesting study about a negative environment and its chronic effects, the withdrawal of environmental enrichments caused barren-housed hens’ comb, face, and eye temperature to drop, and animals performed frustration behaviors for the following 3 weeks. After the same time span, compared to a control group, the experimental group had an increase in corticosterone levels and comb temperature was higher. These changes in parameters might reflect the impact of environmental enrichments and of their withdrawal in barren pens, supporting comb temperature as a long-term marker of stress exposure in laying hens [83]. Another common stressful event in husbandry systems is manipulation. Different studies were carried out on the thermic variation of different body areas of chickens in response to handling [83,84,85]. Handling caused a drop in eye, skin, comb, and wattle temperature [84,85] followed by a post-stressor increase in skin temperature only in side-pinned holding. This is a more stressful hold, with the experimenter holding the chicken to the ground on its side, holding both its legs with one hand, and pushing its side with the other [85]. Interestingly, when analyzing long-term effects, comb, face, and eye temperatures were higher the day after handling [83]. Surface head temperature had a different trend; its temperature rose only after the handling, and the authors suggested that the rise in this region could be representative of the ongoing increase in core body temperature because of ongoing stress-induced hyperthermia, which was, therefore, the cause of peripheral vasoconstriction resulting in reduced comb temperature [84]. Similarly, Moe and colleagues [86] had a person sitting on a chair manually restraining a chick for 10 min. They registered a constant drop in footpad temperature during restraint, while the temperature of all head areas rose with the exception of the comb base. In this case, temperature values were comparable with ‘fight or flight’ mechanisms, with an increase in core temperature and a diminished blood flow in peripheral areas.

Outside husbandry system mechanics, hens’ maternal responses, and reactions to distressed chicks were assessed by separating chicks from mothers and stressing them with air puffs or noises. Hens expressed increased alertness and maternal vocalizations, as well as decreased preening; furthermore, if the chicks were hit by air puffs, hens’ eye and comb temperature decreased [87]. Moe and coworkers [88] examined comb temperature changes following a food reward delivered after anticipation. Temperature dropped after both exposure to the conditioned stimulus and consumption of a reward, but only if the initial temperature was above 30 °C. The drop indicated peripheral vasoconstriction, which should facilitate an increase in core temperature, similar to an emotional fever. Therefore, a drop in comb temperature might be indicative of an emotional arousal more than of an emotional valence, because it occurred in both positive [88] and negative situations [84,85,87].

Concerning wild-living birds, Jerem and colleagues [89] used food to lure blue tits (*Cyanistes caeruleus*) inside a cage trap. Once the animal was inside, a nearby infrared camera measured the baseline value of the eye temperature and immediately after the trap was closed. Upon closure of the trap, eye temperature dropped and reached a minimum, before returning toward baseline over the following 2–3 min; however, it did not reach baseline values by the end of the trial. This was consistent with a reaction to a mild stressor, causing rapid changes in the pattern of blood flow; while the bird remained within the box, eye temperature indicated that the bird remained stressed. Investigating birds’ reactions to danger, Beauchamp [90] compared fowls’ foraging strategies (foraging alone and foraging in pairs) during visual and auditory contact with potential predators; according to the author’s hypothesis, when birds foraged alone, comb, eye, and cheek temperature was lower than baseline, whereas, when birds foraged in pairs, temperature remained stable at baseline values.

## 3. Discussion

By recording electromagnetic radiation normally emitted by whoever or whatever has a temperature superior to absolute zero, infrared thermography can indirectly assess physiological activity occurring when animals react to different emotional stimuli. Unlike other methods, it does not directly affect the studied individuals since it is used remotely, avoiding interference with animals’ behavioral and physiological responses. Infrared thermography is very precise and produces easily handled data. However, despite its apparent ease of use, users have to be very careful during experiments and must take good care of the equipment. Thermographic cameras require calibration before every session because, during the procedure, environmental moisture and temperature are important factors that have to be taken into account, and minor errors in the procedure could result in artefacts that are very difficult to detect and interpret. Rarely, thermographic cameras could, to some extent, be disturbing for animals, causing unexpected behavioral responses [4]. Lastly, it is important to notice that very sensitive thermographic cameras are expensive tools, even if technologic development is making them gradually cheaper.

### 3.1. Physiological Parameters

Differences in recorded temperatures reflect ongoing physiological changes of the animals before, during, and after an emotional event. Physically, the main thing responsible for the temperature recorded with infrared thermography is blood, with both vasodilation and vasoconstriction playing important roles in modifying temperature in different body areas. In particular, in arteriovenous anastomoses (AVAs), which are connections between arterioles and venules bypassing capillary beds, stress-induced vasoconstriction effects are most pronounced. AVAs are present in many different extremities in different taxa, such as primate faces [35], rat tails, rabbit ears, and chicken combs and wattles [85]. Changes in the balancing between the sympathetic and parasympathetic systems are the main cause of vasodilation and vasoconstriction, but it is important to note that there is no rigid opposition between the two branches of the ANS, as the parasympathetic nervous system is active during both pleasant and unpleasant emotions [91].

#### 3.1.1. Nonhuman Primates

In nonhuman primates, nasal temperature changes in response to different emotional states and it could be sensitive to the intensity of the emotion. Thermal imaging experiments showed that it provides a very reliable indicator due to the large presence of arteriovenous anastomoses in the facial region that makes blood flow variation effects more evident [33,35]. However, it is important to control for potentially confounding factors such as locomotor activities, environmental temperature, and, if food is available ad libitum, consumption of a large amount of food [36]. Many different brain areas respond to audiovisual species-specific expressions (e.g., amygdala, ventrolateral prefrontal cortex, superior temporal sulcus, and auditory cortex); however, among those, the amygdala has a key role in emotional responses, memory, and decision-making processes [92], and it is related to arteriovenous anastomoses in the nasal region in primates that are constricted by sympathetic nerves, which suggests that its action might elicit decreases in nasal skin temperatures [35]. Increased adrenergic activity may also be responsible for increases in lip temperature in monkeys and apes during negative emotional states [38].

However, when positively stimulated, nose tip temperatures dropped in both monkeys and apes, while periorbital temperature increased. The drop in the nasal temperature might have the same origin as previously discussed (i.e., arteriovenous anastomosis and cutaneous vasoconstriction), while it is possible to attribute part of the cooling to an increased breathing rate. Increases in the periorbital area might be due to an increased heart rate, which led to increased blood flow to extraocular muscles [33]. It is interesting to note that different facial areas had different reactions to positive emotional stimuli, suggesting different physiological processes involved for the same emotion and likely different amounts of time required to be induced by different emotions [38].

#### 3.1.2. Even-Toed Ungulates

Pre-slaughter analyses in pigs have shown that high blood concentrations of cortisol and creatine kinase correlate with ear temperature values. Because their elevated concentrations indicate high levels of stress, this suggests that hotter pigs might experience more stress [40]. High ear temperature is probably due to an attempt to reduce internal temperature by vasodilation, promoting loss of heat from the ear surfaces [41]. Unlike acute stress, long-lasting stressful stimuli seemed to cause a decrease in ear temperature, likely because of a vasoconstriction effect [42]. Similar to ears, the lacrimal caruncle region reacted to stressful situations, and blood was diverted from cutaneous capillary beds via sympathetically mediated vasoconstriction, provoking a rapid drop in eye temperature. If the stressor persisted for a more than few minutes, cortisol release induced by the HPA axis caused several thermogenic reactions in tissue metabolism [42].

Although dairy cows are highly habituated to human contact, facilities, and procedures, they may still perceive handling as stressful, causing eye temperature to rise [21]. In the case of disbudding, even if preparation procedures are perceived as stressful (causing eye temperature to raise), immediately after disbudding without local anesthetic, there was a general diminution in eye temperature and cutaneous vasoconstriction, probably to avoid possible blood loss. After that, eye temperature increased over baseline values. It has been hypothesized that these reactions may be a sympathetically mediated response as supported by cardiac data in calves, which showed a decrease in heart rate variability parameters, indicating a change in vagal tone and a possible increase in sympathetic activity. It has also been investigated whether these changes could depend on HPA axis activity by exposing the same animals that responded to disbudding to an ACTH challenge, but there were no changes in eye temperature, confirming that eye temperature responses were not a result of HPA axis activity [29,45].

Under stressful conditions, Cannas and colleagues suggested that changes in temperature in the small areas present around the posterior border of the eyelid and the *caruncula lacrimalis* are due to changes in blood flow caused by the activation of the autonomic nervous system, because this area has rich capillary beds innervated by the sympathetic system [47]. On the other hand, Bartolomé and colleagues stated that the increase in eye temperature after a fearful stimulus could be a response associated with the activation of the hypothalamic–pituitary–adrenal axis [48].

#### 3.1.3. Odd-Toed Ungulates

Competition horses experience an activation of the HPA axis and an increase in sympatho-adrenal activity during races, indicated by a rise in heart rate values, which is considered a reliable tool in the study of stress reactions in horses. Temperature and HR show significant correlations both before and just after competitions, thus suggesting a similar physiological basis for both parameters during stressful situations [50]. On the other hand, during grooming activities, temperature and heart rate present a reversed correlation, indicating a parasympathetic control of temperature [93]. A study by Fenner and colleagues [55] reported that eye temperature increased in response to a stressful stimulus, but data did not correlate with heart rate (increment) or HRV (decrement), suggesting different reaction times for the cardiac values and eye temperature. Temperature changes are also influenced by various factors, such as age, habituation, and handler [49,58]. Age and habituation mitigate temperature rises, while a calm, competent, but unknown handler may be equally effective as the owner in managing stressful procedures for the horse [58]. The effects of age and habituation have importance when considering racehorses, because racing performances are influenced by the temperature; it has an elliptical behavior during the race, increasing to a point from which the animals would suffer the consequences of distress, diminishing its performance, and worsening the result. Habituation is the effect of activation of sympatho-adrenal axis and HPA during normal exercise activities, resulting in an acute eustress response. Therefore, healthy and habituated horses have faster recover time, once the stimuli from the competition day are over, while maintaining these stimuli for a prolonged time can cause negative effects with the animal not being able to cope with the stress experienced during the exercise [51].

#### 3.1.4. Rodents and Lagomorphs

In laboratory rodents, it is common to see the effects of fear on skin and eye temperature in various tests, such as the open field or elevated plus maze. Skin temperature depends on blood flow and, thus, on sympathetic vasoconstrictor tone in skin arteries, and studies have shown that fear has a strong effect in lowering temperature, albeit limited to extremities. The tail has a particularly important role in dissipating heat and showing a rebound effect, rapidly raising its temperature after the fearful event, because of its richness of arterio-venous anastomoses. On the other hand, there is only a general warming in the rest of the body [27,61,63]. Eye temperature rises during fearful events, often being the highest temperature of the body when scanning the whole individual with infrared thermography, because of the notable extension of capillary beds surrounding the eyes. Changes in temperature are often associated with behavioral changes, as more reactive or anxious animals have a stronger increase in eye temperature. Interestingly, initial temperature can be used to predict the behavioral responses occurring during the tests. A higher initial temperature of the tail, the eye, and the whole body translates to more intense exploratory and physical activities. However, this means that more active individuals would also be more active in their cages, explaining their high initial body temperatures [61,63]. In response to rearing deprivation, sex differences in hormonal outputs highlighted by Faraji and Metz indicated that females’ HPA axis is initiated more rapidly and remains elevated for longer compared to that of males. Alternatively, these sex-dependent thermal differences may be due to a failure of adaptive responses to acute stress in males or they may reflect females’ vulnerability to the transient vertical-activity deprivation [64].

#### 3.1.5. Carnivores

Stressful situations can induce an increase in core body temperature, which influences the temperature of the lacrimal caruncle and ear pinnae. Veterinary visit caused a peak in dogs’ eye temperatures during the clinical examination phase; on the other hand, temperature of the ear pinnae decreased during isolation and increased when a person was present in the same room with the dog, indicating that isolation stress is associated with reduced ear temperature [4,79]. Similarly, arousal determined by the presence of food in the owner’s hands resulted in an increment in eye temperature [78]. Likely, temperature changes following a stimulus are linked to the intensity of the response, for example, suggesting different effects by stressors of different intensity on the temperature both during and after the exposure, but they cannot be used as the sole parameter for detecting the valence of a stimulus [77,78,79]. This contrasting effect on body temperature can be explained by the activation of the sympathetic branch of the ANS, which induces an increase in core temperature, reflected in the eye, and a decrease in more peripheral body areas, such as the nose, face, and ears, due to vasoconstriction [78]. The HPA axis is especially sensitive to psychogenic stressors and its effects on metabolism, and, along with peripheral vasodilation due to parasympathetic activation during freezing responses, it may explain the increase in eye temperature detected by thermography when dogs were on the examination table [4]. This is in line with the prediction that the stress response through activation of the sympathetic nervous system results in peripheral vasoconstriction, leading to a decrease in surface temperature of the extremities [79].

Interestingly, personality seems to play an important part when dogs have to respond to pain during recovery from a routine castration surgery. Core temperature was significantly lower than control readings, and this situation lingered for more than 3 h after surgery, and this is consistent with what happens with cattle [29]. However, subjects with more extroverted personality had higher peak pain scores and an increase in core temperature, whereas those with a less extroverted personality had a decrease in temperature. Assuming that arousal results in an increase, and pain results in a decrease in temperature, the authors suggested that more extraverted individuals have increased arousal in response to the same tissue damage while introverted individuals experience a pain-induced depression in core temperature [74].

#### 3.1.6. Gallinaceous and Passerine

In hens, body areas of interest for temperature changes, which are easily detected, are the comb, wattle, face, eyes, and footpads.

Both in stressful conditions and in positive situations, a drop in comb temperature has been observed [84,85,87,88]. Restraint causes a significant drop in comb, wattle, and footpad temperatures in broiler chickens [85,86]. Eye temperature decreased during mildly stressful events (air puffs) and increased during more severe events (manual restraints). It is notable that drops in eye temperature occurred even in brooding hens when hearing chicks experiencing a mild stressful event, i.e., an air puff [86,87].

Generally, stress-induced hyperthermia (SIH) causes vasoconstriction in the peripheries to redistribute blood to internal important organs and to prevent blood loss owing to potential injuries, and this causes skin temperature changes, especially in areas with a higher density of arteriovenous anastomoses (AVAs) such as the comb and wattle. Blood flow through AVAs increases in proportion to core body temperature, and temperature changes observed in these regions suggest a link between skin surface patterns and stress-induced hyperthermia [84,85,87]. 

The experiment of Moe and colleagues [88] showed that using emotional fever as an indicator of welfare has limitations as it is difficult to interpret as an indicator of positive or negative emotional valence. However, Herborn and colleagues [83] studied the long-term effects of stress and they reported that withdrawal of enrichments and intermittent handling caused a drop in comb, face, and eye temperature after 2–2.5 h. This was followed by a prolonged elevation in face and eye temperature in the subsequent days, suggesting that skin warming could be used as an indicator of past acute stressful events.

### 3.2. Infrared Thermography Evaluation

As seen so far, different fields have diffuse applications of infrared thermography, and, regarding animals’ emotions, IRT is usable in a wide range of species. Therefore, there is consensus about its versatility, reliability under field conditions, costs to purchase and to operate, and instant access to data that can be useful both for research purposes and for animal management in husbandry systems.

In different experiments and species, IRT assesses temperature changes when scanning different body parts; therefore, target areas should be easily accessible without stressing or scaring animals and without heavy coverage of fur or plumage. For example, in cattle and sheep, eye temperature can detect acute responses of fear or pain, and it can be more immediate and detailed than HPA activity [29,45,94]. Temperature changes in the nose can be used reliably to measure respiratory rate remotely in dairy cows [46]; in macaque monkeys, they occur in response to audiovisual stimuli more than skin conductance responses, suggesting their usefulness in distinguishing emotional states, even if they happen more slowly than conductance responses [35]. Additionally, the nasal region has clear visibility, allowing highly reliable tracking [39]. The comb, eye, and head showed temperature changes in response to handling and other husbandry procedures in chickens, with the largest change occurring in the comb [84]. Bare skin temperature can help in evaluating effects of environmental enrichment and social structure in weaned piglets [42] or acute stress intensity in birds, in which species-specific validations of optimal skin regions are still needed and age is an important factor [85]. Moreover, in laboratory mice, when accounting for missing value frames, as well as intra-rater and inter-rater agreement, the mean body temperature value is considered to be the best for measuring thermal variations [65]. Skin temperature changes can be rapid; thus, measurements should be taken at intervals significantly shorter than the expected speed of response and interpreted in relation to the exact timing of the measurement [85]. In horses, it is also important to note that eye temperature returned to normal values after stimulation faster than HR and HRV, meaning that temperature could be a more sensitive measure [55]. Lastly, in racing dogs, the lacrimal caruncle was the location with the highest sensitivity to temperature changes after races [76].

Keeping in mind that individual experiences might vary how animals cope with different events, there is evidence that temperature changes depend on stimulus intensity, while more experimental evidence is needed to clearly define the effect of stimulus valence on surface temperature changes. Additionally, it is important to notice that some emotional states may not involve marked changes in arousal [95]. In macaques, an audiovisual stimulus induced stronger changes in nasal skin temperatures than did a visual or auditory stimulus alone [35]. Racehorses’ temperature is a suitable tool to assess stress levels during competition and the level when this stress becomes distress, compromising welfare, but the authors noted that measured stress is partly influenced by the horse’s stress susceptibility and temperament [51]. Furthermore, horses are able to dissimulate their condition, as their stress behavior may not correlate with cardiac or thermal parameters; therefore, the behavioral indicators commonly used by the equestrian industry may not be reliable indicators of an animal’s ability to tolerate a stressing event [59]. Moreover, temperature responses seem to be influenced by age and genetic line [50,96]. Similarly, in pigs, thermal responses reflect emotional reactions to acute stressors, as well as other physiological and metabolic indicators, but the relationship between peripheral temperature and individual differences in stress responses needs further investigation [43]. Furthermore, in some cases, results showed that infrared thermography is suitable for detecting a state of arousal but not for assessing the hedonic values of a positive stimulus, unless combined with other indicators, such as skin conductance responses, cortisol concentration, and behavior [78]. However, when using different physiological indicators, the reaction time of each to the stimulus has to be taken into consideration (e.g., HR reacts faster than eye temperature [77]). 

Results that are more consistent were obtained with highly arousing negative stimuli, likely because they are perceived in the same way by all individuals. On the other hand, it is more difficult to identify a universally positive stimulus. Therefore, measurement of valence independently of arousal continues to be difficult [39], with other sources of stress that have to be minimized or carefully recorded prior to exposure to the desired stress stimulus; animals should also have enough time to recover between two stressors to prevent any carryover effects. Moreover, given the rapidity of the response, baseline values should be measured in situ, prior to any data collection [85].

Infrared thermography has some potential limitations that require some precautions when collecting data. Individual-level studies require individual baseline measurements; it has been found that inter-individual temperatures vary consistently, and pre-stressor temperature influenced the magnitude of the temperature response [85]. Because stressors affect the thermal response, data collection should be performed after the animal has settled down into the environment and has become used to the experimenter. In particular, in wildlife fauna, individuals’ responses to environmental changes are hardly predictable [97]; therefore, it is necessary to carefully evaluate effects in the context of skin temperature even when measuring the baseline, to be sure that it is actually a baseline [89]. Furthermore, in at least one study, animals exhibited an avoidance reaction when the thermographic camera was directly oriented toward their muzzle [4], and human presence before the stimulus affected thermal responses in chickens [86]. 

Physical activity causes heat to be dissipated by skeletal muscles, increasing outer body surface temperature [34], and even resting positions have to be considered, because animals resting on their side on cool or moist ground will have cooler areas in the thermogram [97]. Furthermore, in horses, Jansson and colleagues did not find a correlation between maximal eye temperature and rectal temperature [96], while McFarland and colleagues confirmed that surface temperature is more predictive of environmental temperature rather than core temperature, and it should not be used as an indicator of core temperature in wild primates [98]. In mice, the orexinergic system partially controls thermogenesis, influencing sympathetic responses that may reflect inter-individual differences [61], and it is worth recounting that orexins are peptides present in various vertebrates classes [99]. The thickness, color, and quality of hair or feather coat affect the outer surface body temperature; in fact, a thick fur coat makes it difficult to obtain precise data, whereas animals whose skin is barely covered with hair are the ideal models for thermal imaging studies [100]. Thickness of subcutaneous fat, which insulates the body against heat loss, also affects skin temperature. If possible, skin should be cleaned and dried before measurement [97].

Equally important are environmental factors. Sunlight heats the skin, while rainfall and air movements cool it, thus preventing a reliable analysis of internal temperature; therefore, the most suitable weather is overcast. Atmospheric gases and particles lower atmospheric permeability, leading to the absorption (gases) or dissipation (particles) of infrared radiation emitted by objects [97]. Indoor, there should be neither sources of radiation nor sources of air movement. Additionally, heavy breathing of room air might decrease the nasal skin temperature [34]. Generally, more accurate data are gathered when there is a greater difference between the animal and environmental temperatures [97].

## 4. Conclusions

Stress and pain responses have an influence on body temperature in different animals, and these responses can be monitored during different phases of husbandry processes such as routine handling practices [45,84], housing [43], or transport [40], in order to guarantee high welfare standards. Furthermore, a relationship between stress levels and lean pork meat quality has been found [34]. Therefore, thermal data are a valuable tool for the assessment of animal welfare, and they could be integrated into an automated system for the early detection of ill or severely distressed animals [43,46]. Similarly, in sport competitions, infrared thermography may also become useful for the early identification of horses that are not fit to compete or to continue endurance competitions [53]. There are also correlations between dressage competition performances and high temperature values that suggest a possible selection made on a thermic data basis to improve competition results in the offspring, although more studies and more evidence are still needed to prove IRT as a valuable tool for horse selection according to their physiological stress response [60]. In addition, it has been proven that IRT can be used to remotely measure respiratory rate in cattle by automatic detection using an algorithm of changes in temperature due to air movement through the nostrils [46]. Nevertheless, further research is needed to establish the specificity and sensitivity of infrared thermography, as well as how much the magnitude and type of the stress response are influenced by the nature of the stressors, different individuals’ characteristics, and previous experiences.

IRT could even be useful to assess reactions to different events in case-by-case scenarios, considering potential relationships between ocular temperature discrepancy and lateralized cerebral blood flow; an indicator of possible different personality traits that should be investigated further [74]. Other potential applications concern the degree of arousal experienced by an individual when witnessing other individuals’ exciting or stress experiences, how individuals make decisions and solve problems in a foraging context, and how different emotions affect social interactions, or if there is evidence of emotional contagion, helping us to understand complex social interactions [36]. Studies on different reactions to stressful or fearful situations could be enhanced with the administration of anxiolytics or anxiogenic substances, which should decrease or increase the thermic responses, respectively, to further explore the link between IRT data and emotional changes [61]. In some animals, masking signs of fear if flight is not possible may be a fitting strategy, during which the outcome is that physiological responses do not match observed behavior. Consequently, behavior may not always be an accurate indicator of whether the animal found a procedure aversive or not [56]. It is also worth noting the similarities between sheep and human eye, associated with the high reactivity to new stimuli, which make sheep an interesting species for studies about fear [47].

At the present time, an aspect that has been understudied is the evaluation of positive situations for animals with infrared thermography. This should be expanded, with the aid of other physiological correlates such as corticosteroids, heart rate, and heart rate variability, to investigate the physiology of positive emotional activation. Furthermore, in wildlife surveys, IRT enables the acquisition of data that cannot be obtained by any other means, and its versatility, ease of use, and relevance of the result overcome the several limitations, previously discussed, that this technique has when applied in a natural environment [97].

All accounted for, infrared thermography is an important, versatile, and reliable method to study physiological responses to emotional changes due to its ease of use, the absence of any direct contact with the animal for tracking the changes in physiological state, the immediacy of the results, and a slow response time that allows better post-stimulus measurements. These characteristics make infrared thermography data useful for animal behavior and welfare studies, in addition to being available for use in practical settings, for animal management in husbandry systems. A further improvement in the immediacy and reliability of this technique could be achieved through the development of software capable of automatic extraction of the desired area temperature from pictures and/or clips. The relatively low cost of the equipment and the diffusion of technical courses to learn how to properly use an infrared thermographic camera will potentially spread the use of this technique both in academic research and in husbandry systems.

## Data Availability

Not applicable.

## Appendix A

**Table A1:** Characteristics of the sample and the stimulus.

Study (Year)	Species	Sample Size	Stimulus
Bartolomé et al. (2013) [50]	Spanish Sport horse (*Equus ferus caballus*)	86 mares and 87 stallions	Final of a show jumping competition
Bartolomé et al. (2019) [48]	Blanca Serrana goat (*Capra aegagrus hircus*)	10 goat kids, unknown sex, and 14 adult females	Person walking around and within the animals, mak- ing noise and waving arms, while guiding the animals to another pen
Beauchamp (2019) [90]	Chicken	12 hens	Foraging alone or in pairs
Boileau et al. (2019) [43]	Pig (*Sus scrofa domesticus*)	13 weeks old, 17 female and 29 males	Agonistic dyadic encounters
Cannas et al. (2018) [47]	Sarda sheep (*Ovis aries*)	5 ewes	Voluntary animal approach test before and after being restrained
Chotard et al. (2018) [38]	3 common marmosets (*Callithrix jacchus*), 1 white-throated capu- chins (*Cebus capucinus*), 3 rhesus macaques (*Macaca mulatta*), 1 Bor- nean gibbon (*Hylobates* *muelleri*), and 2 western lowland gorillas (*Gorilla gorilla gorilla*)	10 adults, unknown sex	Play with a toy, tickling, food delay, or food teasing
Dai et al. (2015) [57]	Horse	16 mares, 4 stallions, and 30 geld- ings	Unknown noisy item test
Dezecache et al. (2017) [37]	Eastern chimpanzee (*Pan troglodytes* *schweinfurthii*)	3 adult females and 11 adult males	Conspecific vocalizations (screams/whimpers, pant hoots, aggressive barks, travel and resting hoos, grunts, and pant-grunts)
Duparcq et al. (2019) [63]	Mound-building mouse (*Mus spicilegus*)	24 juvenile females and 35 juvenile males	Open field test, novel object test, thermal-response test
Edgar et al. (2011) [87]	Light Sussex and Cots- wold Legbar chicken (*Gallus gallus domesticus*)	32 hens	Separation from the chicks, then an air puff to chicks, an air puff to hens, or a noise of an air puff
Edgar et al. (2013) [84]	Hubbard JA57 chicken	19 hens	Handled by experimenter
Elias et al. (2021) [76]	Greyhound dog (*Canis lupus familiaris*)	465 adults, unknown sex	Race meeting
Ermatinger et al. (2019) [39]	Common marmoset	9 adult females and 8 adult males	Preferred food, playback of food calls, playback of aggressive vocalizations, and food teasing
Faraji and Metz (2020) [64]	Domestic mouse (*Mus musculus domesticus*)	6 adult males and 8 adult females	Restraint and deprivation of rearing during vertical exploration
Fenner et al. (2016) [55]	Horse	2 mares, 3 stallions, and 7 geldings	Noseband tightening
Foster and Ijichi (2017) [80]	Cat (*Felis catus*)	18 females (2 juvenile and 16 adult) and 16 males (2 juvenile and 14 adults)	None. Relations between eye temperature, personal- ity, age, and single/group housing
Fukuzawa et al. (2016) [77]	Dog	9 adult females and 4 adult males	Approached by familiar or stranger human, with a smiling expression or expressionless
Gjendal et al. (2018) [65]	Domestic mouse	6 weeks old, 80 males	Anesthesia with isoflurane for 10 min, handling by scruffing, or intraperitoneal injection of 0.2 mL of 0.9% saline
Herborn et al. (2015) [85]	Lohmann Brown chicken	100 pullets	Handled by experimenter (cradled or side-pinned)
Herborn et al. (2018) [83]	Lohmann Brown chicken	16 weeks old, 56 pullets	Withdrawing environmental enrichments (chronic stress) and intermittent routine handling (acute stress)
Ijichi et al. (2018) [58]	Horse	20 mares and 26 geldings	Novel handling tests, with the owner and with a stranger
Ioannou et al. (2015) [33]	Rhesus macaque	2 females (1 juvenile and 1 adult) and 3 males (1 juvenile and 2 adults)	Play with a toy, food teasing, and feeding sessions
Jaén-Téllez *et al.* (2020) [67]	Spanish rabbit (*Oryctolagus cuniculus*)	40 juveniles, unknown sex	Handled by experimenter
Jerem et al. (2015) [89]	Blue tit (*Cyanistes caeruleus*)	9 adults, unknown sex	Being captured in a closed box
Kano et al. (2016)—experiment 1 [36]	Chimpanzee (*Pan troglodytes*)	4 adult female, and 5 adult males	Audio files of fighting vocalizations by groupmate in- dividuals (screams and barks), display calls by an allo- specific (orangutan) individual, or no sound
Kano et al. (2016)—experiment 2 [36]	Chimpanzee	3 adult females	Video files of conspecific fighting (with screaming and barking vocalizations), conspecific resting (with natu- ral background noises), or blank screen (no sound)
Kuraoka and Nakamura (2011)—experiment 1 [35]	Rhesus macaque	3 adult males (subjects A, B, and C)	Clip of a raging, unfamiliar individual
Kuraoka and Nakamura (2011)—experiment 2 [35]	Rhesus macaque	4 adult males (subjects C, D, E, and F)	Clips of an aggressive threat, a scream, or a ‘coo’ (typi- cal social call)
Kuraoka and Nakamura (2011)—experiment 3 [35]	Rhesus macaque	3 adult males (subjects C, D, and E)	Macaques faces or voices representing a threat
Lecorps et al. (2016) [61]	Domestic mouse	26 adult males	Open field test and elevated plus maze test
Lecorps et al. (2019) [70]	Domestic mouse	16 adult males	Exposure to TMT (2,5-dihydro-2,4,5-trimethylthia- zole), a compound found in the smells of predators
Ludwig et al. (2007) [66]	Rabbit	6 individuals, unknown age and sex	A second individual in the cage, a sudden noise, being placed on the back
Lush and Ijichi (2018) [74]	Dog	20 males, unknown age	Castration
McGreevy et al. (2012) [54]	Horse	3 mares, 1 stallion, and 1 gelding	Wearing a double bridle with and without a cavesson noseband
Moe et al. (2012) [88]	Lohmann Brown Se- lected Leghorns chicken	5 hens	Anticipation and consumption of a palatable reward
Moe et al. (2017) [86]	Ross 308 chicken	30 days old, 20 individuals, un- known sex	Manually restrained by experimenter
Nakayama et al. (2005) [34]	Rhesus macaque	4 adult females	A person standing 1 m away
Negro et al. (2018) [51]	Spanish Trotter horse	46 mares, 71 stallions, and 13 geld- ings	Trotter races
Redaelli et al. (2019) [53]	Arabian horse	4 mares and 4 geldings	Endurance race training
Riemer et al. (2016) [79]	Dog	3 adult females and 3 adult males	Separation from the owner
Rigterink et al. (2018) [72]	Dog	13 females and 33 males	Exposure to unfamiliar people in consultation room of a veterinary hospital
Sánchez et al. (2016) [60]	Pura Raza Español horse	343 stallions	Dressage competition
Schraft and Clark (2017) [68]	Merriam’s kangaroo rat (*Dipodomys merriami*)	27 adult females and 32 adult males	Staged encounter with a predator, Mojave rattlesnake (*Crotalus scutulatus*)
Squibb et al. (2018) [59]	Horse	20 mares and 26 geldings	Navigation toward two distinct obstacles handled by the owner or a stranger
Starling et al. (2020) [75]	Greyhound dog	525 adults, unknown sex	Race meeting
Stewart et al. (2007) [21]	Holstein–Friesian cat- tle (*Bos taurus*)	6 cows	Five exogenous treatments via jugular catheter (saline solution, adrenocorticotropic hormone, bovine cortico- trophin-releasing hormone 20 μg and 40 μg, and epi- nephrine) and a social stimulus (isolation)
Stewart et al. (2008) [29]	Holstein–Friesian cattle	30 female calves	Disbudding with or without anesthetic
Stewart et al. (2008) [45]—experi- ment 1	Hereford × Friesian cattle	6 heisters	Brief slaps on the rump with a plastic tube and brief shakes of a plastic bag in front of the head
Stewart et al. (2008) [45]—exper- iment 2	Cattle	32 bulls	Restraint only, brief applications of an electric cattle prod to the rump area, or brief and sudden shakes of a plastic bag in front of the head accompanied by a loud shout
Stewart et al. (2010) [44]	Friesian cattle	16 male calves	Jugular infusion of either epinephrine or saline solu- tion
Stewart et al. (2017) [46]	Friesian and Friesian × Jersey cattle	22 cows	Restraint in a crush
Travain et al. (2015) [4]	Dog	9 adult females and 5 adult males	Standardized veterinary examination
Travain et al. (2016) [78]	Dog	8 adult females and 11 adult males	Administration of palatable treats
Valera et al. (2012) [49]	Spanish Sport horse	10 mares and 6 males	Final of a show jumping competition
Vianna and Carrive (2005) [27]	Laboratory rat (*Rattus norvegicus domestica*)	12 adult males	Conditioned to a foot shock chamber with or without shock, then exposed again to the foot shock chamber
Warris et al. (2006) [40]	Pig	384 adults, unknown sex	Journey to slaughterhouse
Warris et al. (2006) [41]	Pig	417 adults, unknown sex	Journey to slaughterhouse in trucks with ventilated pens or pens with fan-assisted ventilation
Yáñez-Pizaña et al. (2019) [42]	Pig	64 piglets, unknown sex	Different environmental enrichments (suspended ropes, aromatized bottles, and pet toys and balls) with or without disruption of the social order
Yarnell et al. (2013) [56]	Horse	4 mares and 6 geldings	Sham clipping (running clippers, without active blades)
